# Genetic Characterization of Advance Bread Wheat Lines for Yield and Stripe Rust Resistance

**DOI:** 10.1021/acsomega.3c01981

**Published:** 2023-07-11

**Authors:** Israr Ud Din, Salman Khan, Fahim Ullah Khan, Majid Khan, Muhammad Nauman Khan, Aqsa Hafeez, Sana Wahab, Nazima Wahid, Baber Ali, Umair Bin Qasim, Fazal Manan, Mona S Alwahibi, Mohamed S Elshikh, Sezai Ercisli, Ebaa Mohamed Ali Khalifa

**Affiliations:** † The University of Agriculture Peshawar, Institute of Biotechnology and Genetic Engineering, Peshawar 25130, Pakistan; ‡ Department of Biotechnology, Abdul Wali Khan University Mardan, Mardan 23200, Pakistan; § Department of Agriculture, Hazara University, Mansehra 21120, Pakistan; ∥ Department of Botany, Islamia College Peshawar, Peshawar 25120, Pakistan; ⊥ University Public School, University of Peshawar, Peshawar 25120, Pakistan; # Department of Plant Sciences, Quaid-i-Azam University, Islamabad 45320, Pakistan; ∇ Department of Plant Breeding & Genetics, The University of Agriculture Peshawar, Peshawar 25130, Pakistan; ○ Department of Plant Pathology, North Dakota State University, Fargo, North Dakota 58108-6050, United States; ◆ Department of Botany and Microbiology, College of Science, King Saud University, Riyadh 11451, Saudi Arabia; ¶ Department of Horticulture Faculty of Agriculture, Ataturk University, Erzurum 25240, Türkiye; & HGF Agro, Ata Teknokent, Erzurum 25240, Türkiye; ● Agriculture Research Center, Wheat Research Department, Field Crop Research Institute, Giza 3725005, Egypt

## Abstract

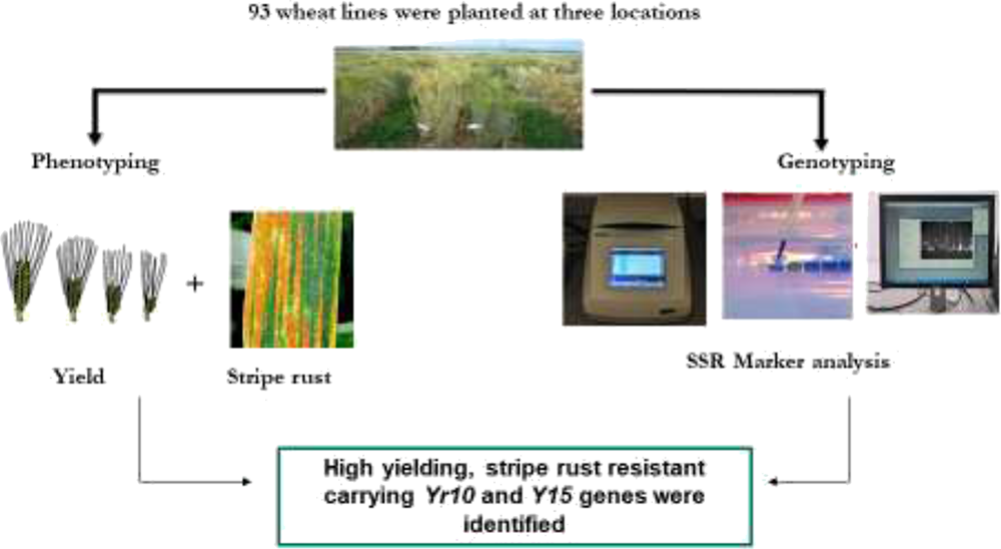

Wheat (*Triticum aestivum* L.) is a prominent grain crop. The goal of the current experiment was to examine the genetic potential of advanced bread wheat genotypes for yield and stripe rust resistance. Ninety-three bread wheat genotypes including three varieties (Kohat-2017, Pakistan-2013, and Morocco) were field tested in augmented design as observational nurseries at three locations (i.e., Kohat, Nowshera, and Peshawar) during the 2018−19 crop season. Various parameters related to yield and stripe rust resistance showed significant differences among genotypes for most of the characters with few exceptions. The analysis of variance revealed significant variations for all the genotypes for all the traits at all three sites with few exceptions where nonsignificant differences were noticed among genotypes. Averaged over three locations, genotypes exhibiting maximum desirable values for yield and yield components were KT-86 (325 tillers) for tillers m^–2^, KT-50 (2.86 g) for grain weight spike^–1^, KT-49 (41.6 g) for 1000-grain weight, KT-50 (74 grains) for grains spikes^–1^, KT-55 (4.76 g) for spike weight, and KT-36 and KT-072 (4586 kg ha^–1^) for grain yield. Correlation analysis revealed that grain yield had a significant positive correlation with grain spike^–1^ and grain weight spike^–1^ at Kohat, with grains spike^–1^, tillers m^–2^, and grain weight spike^–1^ at Nowshera, and with plant height, spike weight, 1000-grain weight, and tillers m^–2^ at Peshawar. Molecular marker data and host response in the field at the adult stage revealed that *Yr15* and *Yr10* are both still effective in providing adequate resistance to wheat against prevalent races of stripe rust. Four lines showing desirable lower average coefficient of infection (ACI) values without carrying *Yr15* and *Yr10* genes show the presence of unique/new resistance gene(s) in the genetic composition of these four lines. Genotype KT-072 (4586 kg ha^–1^ and 1.3 ACI), KT-07 (4416 kg ha^–1^ and 4.3 ACI), KT-10 (4346 kg ha^–1^ and 1.0 ACI), and KT-62 (4338 kg ha^–1^ and 2.7 ACI) showed maximum values for grain yield and low desirable ACI values, and these lines could be recommended for general cultivation after procedural requirements of variety release.

## 1 Introduction

Wheat (*Triticum aestivum* L.) is one of the staple food crops of millions of people around the globe.^1−4^ Wheat provides 21% of global food demand as a regular meal and is produced on over 200 million acres of farmland.^5,6^ Wheat production is influenced by several abiotic factors such as drought,^7−10^ salinity,^11−18^ and heavy metals^19−22^ and biotic factors.^10,23−26^ Biological pests and pathogens cause severe damage to wheat crops, resulting in low production and a loss of 10–50% yield depending on the type of disease and region.^27^ Stripe rust caused by *Puccinia striiformis* is one of the diseases that has affected many countries around the world, including Pakistan.^28^ Wheat stem rust induced by *P. graminisf*. sp. *tritici* affects the stem, whereas wheat leaf rust caused by *Puccinia triticina* affects the leaf, both of which have an impact on yield. When these three rusts are combined, significant wheat loss results, but in Pakistan, specifically in Khyber Pakhtunkhwa, wheat stripe rust is a major factor for decreased production.^29^ Stripe rust (*P. striiformis*) is a fungus in the kingdom fungi that belongs to the phylum Basidiomycota.^30^ Stripe rust is prevalent in 70% of Pakistan’s wheat cultivation area.^31^ More than $5 billion is lost to cereal rusts (leaf rust, stem rust, and stripe rust) worldwide each year. Crop damages can reach 50–100% due to infected plants and shriveled grain. Over time, several regions of Khyber Pakhtunkhwa, Punjab, and Baluchistan have seen an increase in the prevalence of stripe rust. Both the existence of a favorable habitat for these infections and the lack of resistance in cultivated cultivars are the primary causes of this degradation.^32^ Creating resistant cultivars is the most crucial economic and environmental measure. Breeders claim that more than 37 genes in wheat stocks have been found to be resistant to stripe rust.^33^

Many molecular markers are available for the molecular identification of genotypic variation.^34^ The variety of wheat and stripe rust genotypes was evaluated using AFLP’s, RAPD, RFLP’s, and sequencing-based characterization.^35^ The application of genetic markers is determined by their physical properties and genomic location, the cost and benefits involved, convenience in practical use, and the extent of throughput needed.^36−39^ The SSRs have preferred DNA-based genetic markers, which are used for genotyping plants over the past 20 years due to their attractive features such as genome-wide coverage, codominant inheritance, highly polymorphic, highly reproducible, and transferability among related species.^40^ Moreover, SSRs can be used by breeders and geneticists for genotyping plants in limited laboratory facilities due to their relatively low cost and easy-to-use tool.^41^

Field-based testing must be carried out across multiple locations to confirm the utility of resistance under field conditions.^42^ This will show how the local environment influences the expression of the underlying genes that resist the pathogen races present.^33^ The creation of wheat cultivars with increased resistance to stripe rust requires both field-based and molecular characterization of the wheat germplasm. A continuous effort is required to develop new varieties with best source of resistance. The current study was designed with the objective to identify high yielding and stripe rust resistance wheat lines based on field scoring and gene specific molecular markers.

## 2 Materials and Methods

In order to screen advanced wheat breeding lines against stripe rust based on field scoring and gene specific molecular markers, a set of 93 lines including 90 advanced wheat lines along with three checks such as Morocco, Kohat-2017, and Pakistan-2013 were planted at Barani Agricultural Research Station, Kohat (E-01), Cereal Crops Research Institute (CCRI), Pirsabak, Nowshera (E-02), and Institute of Biotechnology & Genetic Engineering (IBGE), the University of Agriculture, Peshawar (E-03), during 2018–2019 crop season. All the lab work of the experiments was carried out at IBGE, the University of Agriculture, Peshawar.

### 2.1 Field Layout, Sowing, and Crop Husbandry

A set of 93 genotypes including 90 advanced wheat lines were planted at Barani Agricultural Research Station, Kohat (E-01), Cereal Crops Research Institute, Pirsabak (CCRI), Nowshera (E-02), and IBGE, the University of Agriculture, Peshawar (E-03), in 2018–2019 crop season. Morocco, a supersusceptible wheat variety, was sown around all entries as the spreader as well as to serve as an adult plant susceptible check. Similarly, Morocco (susceptible check), Kohat-2017, and Pakistan-2013 top wheat varieties were added in the experimental trail as check cultivars. The experimental material was planted in 2 small rows with a row length of 1.5 m in augmented design (nonreplicated) consisting of three blocks having 33 subplots (in each block three checks and 30 advance lines). A set of checks were comprised in each incomplete block, and each check was repeatedly occurring in all other blocks, while the advance lines were assigned to different blocks.

### 2.2 Agronomic Data

Along with Yellow rust data, data were also recorded for various agronomic/yield related traits. These parameters include tillers m^–2^, spike weight (g), grain weight spike^–1^, grains spike^–1^, 1000-grain weight, and grain yield (kg ha^–1^).

### 2.3 Yellow Rust Scoring

As the selected sites are hot spots for stripe rust, natural infectivity was based upon this, and disease scoring was noted at the peak of rust infection after heading. Data on response and severity of stripe rust were recorded. The rigorousness was observed as the percent of rust infection on plants conferring to the little modification of Cobb scale.^43^ The coefficient of infection (CI) was calculated by multiplying the response value with the intensity of infection in percent.

### 2.4 Molecular Study

#### 2.4.1 DNA Extraction

Fresh leaves (1–2 g) were collected from all the lines for DNA extraction. Then, leaves were crushed one by one using liquid nitrogen. A modified CTAB (cetyltrimethylammonium bromide) method was used for DNA extraction. The DNA isolated was diluted in 1× TBE buffer, and the purity and concentration of extracted DNA were checked using the Nanodrop. The DNA was stored at −20 °C for further polymerase chain reaction (PCR) analysis.

#### 2.4.2 PCR Amplification

Genes linked with stripe rust resistance including *Yr10* and *Yr15* were analyzed through PCR using specific primers as shown in Table 1.

**Table 1:** List of Primers Used in the Study

gene	primer	primer sequence	product size [bp]	reference
*Yr10*	Xpsp3000	forward: GCAGACCTGTGTCATTGGTC	260 bp	Wang et al.^144^
		reverse: GATATAGTGGCAGCAGGATAC		
*Yr15*	Xbarc8-1B	forward: GCGGGAATCATGCATAGGAAAACAGAA	190	Murphy et al.^145^
		reverse: GCGGGGGCGAAACATACACATAAAAACA		

#### 2.4.3 Gel Electrophoresis

After successful completion of PCR reactions, the amplified product was checked on 2% Agarose gel, and the band of different size isolates were compared with the described sizes in original papers reporting development of the markers.

### 2.5 Statistical Analysis

Data recorded on each trait was subjected to analysis of variance appropriate for augmented design. The statistical model, separate for each location, is as follows:
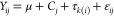
1where *Y_ijk_* = observed value of genotype *i* in block *j*, μ = mean, *C* = checks, τ = new lines, and ε = error.

#### 2.5.1 Pearson-Correlation Coefficients

Pearson-correlation coefficients between each pair of morpho-physio and yield characters were worked out separate for each location through the computer package SPPS v.16.

## 3 Results

### 3.1 Tillers m^–2^

Analysis of variance revealed significant differences among genotypes and lines at all three locations, whereas checks vs lines were found significant at Kohat and Nowshera, while checks were found significant at Kohat only (Table 2). Adjusted mean data for tillers m^–2^ at Kohat, Nowshera, and Peshawar are given in Table 3. Data for tillers m^–2^ ranged from 78 to 307 with a mean value of 177 at Kohat, 95 to 392 with an average value of 250 at Nowshera and 75 to 403 with a mean value of 190 at Peshawar. At Kohat, 38, 54, and 77 out of 90 lines produced more tillers than Pakistan-13, Kohat-2017, and Morocco, respectively. Minimum and maximum values for tillers m^–2^ were produced by KT-26 (78 tillers) and KT-37 (307 tillers), respectively, when compared with check cv. Pakistan-2013 (158 tillers), Kohat-2017 (140 tillers), and Morocco (110 tillers) at Kohat. Similarly, at Nowshera, 25, 26, and 42 out of 90 lines produced more tillers than Morocco, Pakistan-13 and Kohat-2017, respectively. Minimum and maximum values for tillers m^–2^ were recorded for KT-26 (95 tillers) and KT-19 (392 tillers) when compared with check cv. Morocco (221 tillers), Pakistan-2013 (215 tillers), and Kohat-2017 (172 tillers) at Nowshera. Likewise at Peshawar, although checks vs lines contrast were found nonsignificant for tillers m^–2^, minimum and maximum values for tillers m^–2^ were observed for KT-09 (75 tillers) and KT-05 (403 tillers) when compared with check cv. Pakistan-2013 (187 tillers), Kohat-2017 (178 tillers), and Morocco (142 tillers) at Peshawar (Supplementary Table S1).

**Table 2:** Means Square for Various Traits of 93 Wheat Genotypes at Kohat, Nowshera, and Peshawar during 2018–2019

traits	Kohat					Nowshera					Peshawar				
	genotypes (df = 92)	lines (*L*) (df = 89)	checks(*C*) (df = 2)	*L* vs *C* (df = 1)	error (df = 6)	genotypes (df = 92)	lines (*L*) (df = 89)	checks (*C*) (df = 2)	*L* vs *C* (df = 1)	error (df = 6)	genotypes (df = 92)	lines (*L*) (df = 89)	checks (*C*) (df = 2)	*L* vs *C* (df = 1)	error (df = 6)
tillers m^–2^	2599.3**	2480.3**	1781.3**	14819.2**	60.9	8017.1^**^	7910.6^**^	2103.5^NS^	29322.8^**^	884.2	3520.6*	3555.5*	1755.7^NS^	3943.6^NS^	787.6
spike weight	1.11^**^	1.05^**^	0.67^**^	7.33^**^	0.04	0.37*	0.30^NS^	2.49^**^	1.77^**^	0.09	0.32*	0.31*	0.65^**^	0.42*	0.07
grain weight spike^–1^	0.206^**^	0.203^**^	0.455^**^	0.009^NS^	0.003	0.224*	0.208^NS^	0.765^**^	0.490*	0.062	0.190^NS^	0.191 ^NS^	0.138 ^NS^	0.250 ^NS^	0.070
grains spike^–1^	111.71^**^	110.25^**^	99.20^**^	266.66^**^	4.52	170.41^**^	169.23^**^	78.27^NS^	459.77^**^	24.09	105.02^NS^	106.55 ^NS^	87.26 ^NS^	4.34 ^NS^	41.65
1000-grain weight	13.26^**^	10.07^**^	45.51^**^	233.21^**^	0.40	32.57^**^	3.06^NS^	702.82^**^	1318.71^**^	1.07	12.99*	8.65^NS^	167.92^**^	88.66^**^	3.23
grain yield	914,282^**^	895,228^**^	2,155,405^**^	127,841*	12,743	944,196^**^	864,580^**^	564,406^**^	8,789,571^**^	40,910	591,074^**^	580,331^**^	1,364,637^**^	135^NS^	77,762

**Table 3:** Descriptive Statistics of 93 Wheat Genotypes for Various Traits across Three Locations

environment	parameter	TLR	SW	GWS	GSPK	TGW	GY
across environments	range	75–403	1.57–6.93	0.25–3.69	10–89	29.8–61.2	1328–5758
	mean	206	3.53	1.94	51	37.8	3402
	desirable gen.	KT-86	KT-55	KT-50	KT-50	KT-49	KT-36, KT-072
Kohat (E-1)	range	78–307	2.73–6.93	1.21–3.69	26–89	32.7–49.5	1417–5758
	mean	177	4.03	2.06	49	42.3	2909
	desirable gen.	KT-37	KT-46	KT-60	KT-050	KT-37	KT-58
Nowshera (E-2)	range	95–392	1.57–4.27	0.25–2.95	10–87	30.8–61.2	2927–5671
	mean	250	2.92	1.74	50	34.8	4238
	desirable gen.	KT-19	KT-40	KT-51	KT-51	KT-18	KT-061
Peshawar (E-3)	range	75–403	2.28–5.08	1.07–3.07	34–86	29.8–46.3	1328–5379
	mean	190	3.63	2.01	55	36.3	3058
	desirable gen.	KT-05	KT-01	KT-60	KT-02	KT-51	KT-05

### 3.2 Spike Weight (g)

Mean squares for spike weight showed significant differences among genotypes, checks, and checks vs lines at all three locations, whereas lines were found significantly different at Kohat and Peshawar only (Table 2). Adjusted mean data for spike weight at Kohat, Nowshera, and Peshawar are given in Table 3. Data for spike weight ranged from 2.73 to 6.93 g with a mean value of 4.03 g at Kohat, 1.57 to 4.27 g with an average value of 2.92 g at Nowshera, and 2.28 to 5.08 g with a mean value of 3.63 g at Peshawar. At Kohat, 27, 62, and 62 out of 90 lines produced heavy spikes than check cv. Kohat-2017, Pakistan-2013, and Morocco, respectively. Minimum and maximum values for spike weight were noticed for KT-28 (2.73 g) and KT-46 (6.93 g) when compared with check cv. Kohat-2017 (3.66 g), Pakistan-2013 (2.89 g), and Morocco (2.79 g). Similarly, at Nowshera, none of the tested lines were significantly different from check cv. Kohat-2017 and Pakistan-2013 in terms of spike weight. However, 26 out of 90 lines produced heavy spikes than Morocco. Minimum and maximum values for spike weight were recorded for KT-81 (1.57 g) and KT-40 (4.12 g) when compared with Kohat-2017 (4.27 g), Pakistan-2013 (3.39 g), and Morocco (2.45 g) at Nowshera. Likewise, at Peshawar, 9, 9, and 47 out of 90 significantly produced heavy spikes than Kohat-2017, Pakistan-13, and Morocco, respectively. Minimum and maximum values for spike weight were observed for KT-026 (2.28 g) and KT-01 (5.08 g) when compared with Kohat-2017 (3.69 g), Pakistan-2013 (3.68 g), and Morocco (2.88 g) at Peshawar (Supplementary Table S2).

### 3.3 Grain Weight Spike^–1^ (g)

Data pertaining grain weight spike^–1^ exhibited significant differences among genotypes, lines and checks at Kohat, while genotypes, checks, checks vs lines showed significant differences at Nowshera, whereas nonsignificant differences at Peshawar (Table 2). Adjusted mean data for grain weight spike^–1^ at Kohat, Nowshera, and Peshawar are given in Table 3. Data for grain weight spike^–1^ ranged from 1.21 to 3.69 g with a mean value of 2.06 g at Kohat, 0.25 to 2.95 g with an average value of 1.74 g at Nowshera, and 1.07 to 3.07 g with a mean value of 2.01 g at Peshawar. At Kohat, 61, 10, and 32 out of 90 lines of the tested line produced heavier grain weight spike^–1^ compared with check cv. Morocco, Kohat-2017, and Pakistan-2013. Minimum and maximum values for grain weight spike^–1^ were noticed for KT-39 (1.07 g) and KT-60 (3.07 g) when compared with check cv. Morocco (1.67 g), Kohat-2017 (2.44 g), and Pakistan-2013 (1.97 g) at Kohat. At Nowshera, 16 and 5 out of 90 tested lines produced heavier grain weight spike^–1^ than check cv. Morocco and Pakistan-2013, respectively. However, none of the tested lines produced heavier grain weight spike^–1^ than check cv. Kohat-2017. Minimum and maximum values for grain weight spike^–1^ were recorded for KT-80 (0.25 g) and KT-51 (2.95 g) when compared with check cv. Kohat-2017 (2.33 g), Pakistan-2013 (2.26 g), and Morocco (1.93 g) at Nowshera. At Peshawar, data for grain weight spike^–1^ were nonsignificant, however minimum and maximum values for grain weight spike^–1^ were observed for KT-39 (1.07 g) and KT-60 (3.07) when compared with Pakistan-2013 (2.0 g), Morocco (2.0 g), and Kohat-2017 (3.0 g) (Supplementary Table S3).

### 3.4 Grain Spike^–1^

Data regarding grain spike^–1^ showed significant and nonsignificant differences among genotypes, lines, checks and checks vs lines at Kohat and Peshawar, respectively, whereas genotypes, lines, and checks vs lines at Nowshera (Table 2). Adjusted mean data for grain spike^–1^ at Kohat, Nowshera, and Peshawar are given in Table 3. Data for grain spike^–1^ ranged from 26 to 89 with a mean value of 49 at Kohat, 10 to 87 with an average value of 50 at Nowshera, and 34 to 86 with a mean value of 56 at Peshawar. At Kohat, 6, 15, and 15 out of 90 lines produced more grains spikes^–1^ than check cv. Kohat-2017, Pakistan-2013, and Morocco, respectively. Minimum and maximum values for grain spikes^–1^ were noticed for KT-37 (26 grains) and KT-050 (89 grains) when compared with check cv. Kohat-2017 (61 grains), Pakistan-2013 (51 grains), and Morocco (51 grains). Similarly, at Nowshera, 20, 33, and 36 out of 90 lines produced more grains spikes^–1^ than check cv. Morocco, Kohat-2017, and Pakistan-2013, respectively. Minimum and maximum values for grain spikes^–1^ were noticed for KT-80 (10 grains) and KT-51 (87 grains) when compared with check cv. Morocco (49 grains), Kohat-2017(41 grains), and Pakistan-2013 (39 grains). Likewise, although differences among genotypes at Peshawar were nonsignificant for grain spikes^–1^, minimum and maximum values for grain spikes^–1^ were noticed for KT-39 (34 grains) and KT-02 (86 grains) when compared with check cv. Morocco (61 grains), Pakistan-2013 (56 grains), and Kohat-2017 (51 grains) (Supplementary Table S4).

### 3.5 1000 Grain Weight (g)

Mean squares for 1000-grain weight showed significant differences among genotypes, checks, and checks vs lines at all three locations, whereas lines were found significant at Kohat only (Table 2). Adjusted mean data for 1000-grain weight at Kohat, Nowshera, and Peshawar are given in Table 3. Data for 1000-grain weight ranged from 32.7 to 49.5 g with a mean value of 42.3 g at Kohat, 30.8 to 61.2 g with an average value of 34.8 g at Nowshera, and 29.8 to 46.29 g with a mean value of 36.3 g at Peshawar. At Kohat, 34 and 64 out of 90 lines produced heavy 1000-grain weight than check cv. Kohat-2017 and Pakistan-2013; whereas all the tested lines produced heaviest grain weight than that of check cv. Morocco. Minimum and maximum values for 1000-grain weight were noticed for KT-21 (37.4 g) and KT-37 (39.5 g) when compared with check cv. Kohat-2017 (40.0 g), Pakistan-2013 (38.6 g), and Morocco (32.7 g). Similarly, at Nowshera, none of the tested lines produced heavy grains than check cv. Kohat-2017 and Pakistan-2013; however, 32 out of 90 tested lines produced heavier grain weight than check cv. Morocco. Minimum and maximum values for 1000-grain weight were recorded for KT-73 (31.8 g) and KT-18 (38.8 g) when compared with Kohat-2017 (61.2 g), Pakistan-2013 (49.3 g), and Morocco (30.8 g) at Nowshera. Likewise, at Peshawar, no lines produced heavy grain weight than Kohat-2017 and Pakistan-13; however, 8 lines produced heavier grain weight than Morocco. Minimum and maximum values for 1000-grain weight were observed for KT-88 (29.8 g) and KT-51 (42.2 g) when compared with Kohat-2017 (46 g), Pakistan-2013 (41 g), and Morocco (31 g) at Peshawar (Supplementary Table S5).

### 3.6 Grain Yield (kg ha^–1^)

Mean squares for grain yield showed significant differences among genotypes, checks, and lines at all three locations, whereas checks vs lines were found significantly different at Peshawar only (Table 2). Adjusted mean data for grain yield at Kohat, Nowshera, and Peshawar are given in Table 3. The grain yield ranged from 1417.28 to 5758.02 kg ha^–1^ with a mean value of 2908.62 kg ha^–1^ at Kohat, 2926.85 to 5671.30 kg ha^–1^ with an average value of 4247.58 kg ha^–1^ at Nowshera, and 1328.70 to 5378.70 kg ha^–1^ with a mean value of 3042.54 kg ha^–1^ at Peshawar. At Kohat, 19, 24, and 70 out of 90 lines exceeded check cv. Kohat-2017, Pakistan-2013, and Morocco, respectively. Minimum and maximum values for grain yield were noticed for KT-30 (1417.28 kg ha^–1^) and KT-58 (5758.02 kg ha^–1^) when compared with check cv. Kohat-2017 (3426 kg ha^–1^), Pakistan-2013 (3111 kg ha^–1^), and Morocco (1826 kg ha^–1^). Similarly, at Nowshera, 60, 59, and 77 lines produced higher grain yield than check cv. Kohat-2017, Pakistan-2013, and Morocco, respectively. Minimum and maximum values for grain yield were recorded for KT-80 (1926.85 kg ha^–1^) and KT-061 (5671.30 kg ha^–1^) when compared with Morocco (2739 kg ha^–1^), Pakistan-2013 (3542 kg ha^–1^), and Kohat-2017 (3425 kg ha^–1^) at Nowshera. Likewise, at Peshawar, 8, 8, and 41 out of 90 lines significantly exceeded check cv. Kohat-2017, Pakistan-13, and Morocco, respectively. Minimum and maximum values for grain yield were observed for KT-09 (1328.70 kg ha^–1^) and KT-05 (5378.70 kg ha^–1^) when compared with Kohat-2017 (3453 kg ha^–1^), Pakistan-2013 (3433 kg ha^–1^), and Morocco (2275 kg ha^–1^) at Peshawar (Supplementary Table S6).

### 3.7 Correlation Analysis

**Figure 1:**
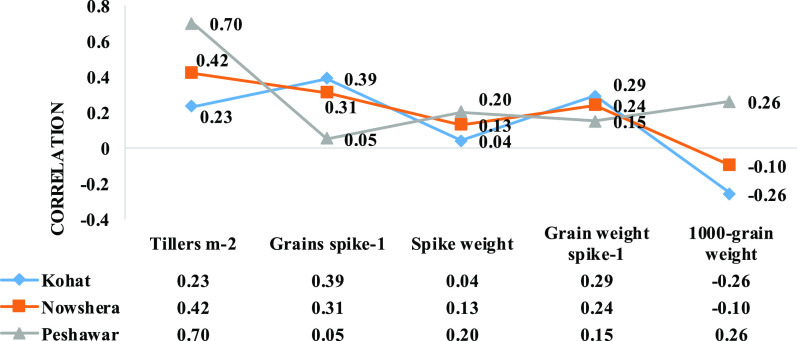
Correlation coefficient among yield and yield components of 93 wheat genotypes at Kohat, Nowshera, and Peshawar during 2018–2019.

Correlation coefficient among morpho-yield traits at Kohat, Nowshera, and Peshawar are given in Figure 1. At Kohat, grain yield showed significant positive correlation with days to maturity (*r* = 0.11), days-to-heading (*r* = 0.40), grain spike^–1^ (*r* = 0.39), and grain weight spike^–1^ (*r* = 0.29), while significantly negative correlation with 1000-grain weight (*r* = −0.26). Likewise, grain yield showed significant positive relationship with grains spike^–1^ (*r* = 0.39), grain weight spike^–1^ (*r* = 0.24), and tillers m^–2^ (*r* = 0.42), whereas significant negative correlation with days to maturity (*r* = −0.24 at Nowshera. Furthermore, grain yield depicted significant positive association with plant height (*r* = 0.33), 1000-grain weight (*r* = 0.26), and spike weight (*r* = 0.20), and tillers m^–2^ (*r* = 0.70) at Peshawar.

### 3.8 Stripe Rust Incidence

Data on host reaction to stripe/yellow rust fungus were noted at the peak stage in all three trials on 93 genotypes at Barani Agricultural Research Station, Kohat, IBGE, Peshawar, and Cereal Crops Research Institute, Pirsabak Nowshera Pakistan during 2018–2019 crop season. Stripe rust scores along with CI of 93 genotypes including 90 advance lines and 3 check cultivars such as Kohat-2017, Pakistan-2013, and Morocco are given in Table 4. Host reaction of all categories was observed, that is, no visible infection (0), resistant (R), moderately resistant-moderately susceptible (M), moderately resistant (MR), moderately susceptible (MS), susceptible (S), and their combinations (MSS). Overall, based on the average CI (ACI), 39 lines including check cv. Pakistan-2013 showed ACI values that varied between 0.3 and 9.3, 45 lines including check cv. Kohat-2017 showed ACI values between 10.7 and 20, whereas 8 lines showed ACI values between 21.3 and 49.3 in comparison with susceptible check Morocco (93.3).

**Table 4:** Stripe Rust Scoring of 93 Wheat Genotypes with Coefficient of Infection (CI) and Average Coefficient of Infection (ACI) at Three Sites during 2018–2019 Crop Season

genotype	Kohat		Nowshera		Peshawar		ACI	*Yr10*	*Yr15*
	YR score	CI	Yr score	CI	Yr score	CI			
KT-01	10MR	4	30M	18	10M	6	9.3	+	+
KT-02	0	0	5MR	2	5R	1	1.0	+	–
KT-03	20MR	8	40M	24	30M	18	16.7	+	+
KT-04	30M	18	10M	6	5R	1	8.3	+	+
KT-05	30M	18	30M	18	20M	12	16.0	–	–
KT-06	30M	18	40M	24	20M	12	18.0	+	+
KT-07	5R	1	10M	6	10M	6	4.3	+	+
KT-08	5R	1	TR	0	0	0	0.3	+	+
KT-09	10MR	4	30M	18	10M	6	9.3	+	+
KT-10	0	0	5MR	2	5R	1	1.0	+	+
KT-11	20MR	8	40M	24	30M	18	16.7	+	+
KT-12	5MR	2	30M	18	40MR	16	12.0	+	+
KT-13	10R	2	20M	12	20M	12	8.7	–	+
KT-14	60S	60	30M	18	40MR	16	31.3	–	–
KT-15	10MR	4	10M	6	10MR	4	4.7	–	–
KT-16	20MR	8	40M	24	30M	18	16.7	+	+
KT-17	10MR	4	20M	12	10MR	4	6.7	+	–
KT-18	40M	24	20M	12	30M	18	18.0	–	–
KT-19	30M	18	10M	6	5R	1	8.3	+	+
KT-20	30M	18	30M	18	20M	12	16.0	+	–
KT-21	30M	18	40M	24	20M	12	18.0	+	+
KT-22	20MR	8	20M	12	20M	12	10.7	+	+
KT-23	30M	18	20M	12	20MR	8	12.7	+	+
KT-24	20MR	8	10MR	4	20M	12	8.0	–	+
KT-25	10MR	4	5M	3	0	0	2.3	+	–
KT-26	30M	18	20M	12	20MR	8	12.7	+	+
KT-27	10MR	4	10MR	4	5R	1	3.0	+	+
KT-28	10MR	4	5MR	2	5R	1	2.3	+	+
KT-29	10MR	4	20M	12	20M	12	9.3	+	+
KT-30	10MR	4	20M	12	10MR	4	6.7	–	+
KT-31	30MS	24	40M	24	20M	12	20.0	–	+
KT-32	10MR	4	10M	6	10M	6	5.3	–	+
KT-33	5R	1	5M	3	0	0	1.3	–	+
KT-34	10MR	4	TR	0	0	0	1.3	–	+
KT-35	30M	18	30M	18	30M	18	18.0	–	+
KT-36	30M	18	40M	24	30M	18	20.0	–	+
KT-37	30M	18	5MR	2	5M	3	7.7	+	+
KT-38	30M	18	10MR	4	10M	6	9.3	+	+
KT-39	20MR	8	30M	18	20M	12	12.7	+	+
KT-40	30M	18	40M	24	30M	18	20.0	+	+
KT-41	20MR	8	30M	18	20M	12	12.7	+	+
KT-42	10MR	4	30M	18	20M	12	11.3	+	+
KT-43	30M	18	30M	18	20M	12	16.0	+	–
KT-44	10MR	4	TR	0	0	0	1.3	+	+
KT-45	30M	18	10M	6	5R	1	8.3	+	+
KT-46	20MR	8	10M	6	5R	1	5.0	–	+
KT-47	10MR	4	30MS	24	20M	12	13.3	–	+
KT-48	30MS	24	5M	3	0	0	9.0	–	–
KT-49	5R	1	5M	3	0	0	1.3	–	–
KT-50	10MR	4	TR	0	0	0	1.3	–	+
KT-51	0	0	10M	6	5R	1	2.3	–	+
KT-52	5R	1	40M	24	30MR	12	12.3	+	+
KT-53	0	0	10M	6	0	0	2.0	–	+
KT-54	5R	1	TR	0	0	0	0.3	–	–
KT-55	0	0	10MR	4	5R	1	1.7	–	+
KT-56	30M	18	30M	18	30M	18	18.0	+	+
KT-57	30M	18	30M	18	30M	18	18.0	+	+
KT-58	20MR	8	40M	24	40M	24	18.7	+	+
KT-59	10MR	4	40M	24	40MR	16	14.7	+	+
KT-60	20MR	8	40M	24	30M	18	16.7	+	+
KT-61	80S	80	20M	12	10M	6	32.7	–	–
KT-62	5R	1	10M	6	5R	1	2.7	–	–
KT-63	20MR	8	40M	24	30M	18	16.7	+	+
KT-64	20MR	8	20MR	8	40MR	16	10.7	+	+
KT-65	40M	24	30M	18	20M	12	18.0	+	+
KT-66	20MR	8	30MS	24	40MS	32	21.3	+	+
KT-67	10MR	4	40MS	32	30M	18	18.0	+	+
KT-68	20MR	8	30M	18	30M	18	14.7	–	+
KT-69	30MR	12	30M	18	30M	18	16.0	+	+
KT-70	30MR	12	30M	18	20M	12	14.0	+	+
KT-71	5R	1	5MR	2	0	0	1.0	+	+
KT-72	5R	1	0	0	5M	3	1.3	+	+
KT-73	40M	24	20R	4	20M	12	13.3	+	+
KT-74	10MR	4	20M	12	10M	6	7.3	+	–
KT-75	20MR	8	50M	30	30M	18	18.7	+	+
KT-76	40M	24	30M	18	20M	12	18.0	+	–
KT-77	40M	24	20M	12	10M	6	14.0	–	–
KT-78	20MR	8	20M	12	10M	6	8.7	+	+
KT-79	5R	1	20M	12	20MR	8	7.0	+	+
KT-80	40M	24	30M	18	20M	12	18.0	+	+
KT-81	40M	24	20M	12	10M	6	14.0	+	+
KT-82	60S	60	60MS	48	50MS	40	49.3	–	–
KT-83	40M	24	30M	18	20M	12	18.0	+	+
KT-84	40M	24	50M	30	30M	18	24.0	+	–
KT-85	40M	24	30M	18	20M	12	18.0	+	+
KT-86	20MR	8	30M	18	30M	18	14.7	–	+
KT-87	40MSS	36	40MS	32	30MS	24	30.7	–	–
KT-88	60S	60	50M	30	30M	18	36.0	–	–
KT-89	30M	18	40M	24	20M	12	18.0	–	–
KT-90	60S	60	60MS	48	50MS	40	49.3	–	–
Kohat-17	40M	24	30M	18	20M	12	18.0	–	+
Pakistan-13	20MR	8	20M	12	10M	6	8.7	+	–
Morocco	90S	90	100S	100	90S	90	93.3	–	–

Among 93 genotypes, five lines showed 0-TR reaction with CI values of 0, 39 lines showed MR reaction with CI values between 5 and 12, 11 lines showed R type of reaction with a CI value between 1 and 2, 29 lines exhibited MR-MS or M reaction with CI values between 18 and 24, 2 lines showed MS reaction with a CI value of 24, one line showed MSS reaction with a CI value of 36 and 6 lines, including Morocco expressed S reaction with a CI value between 60 and 90 at Kohat. Similarly, at Nowshera, 6 lines exhibited 0-TR reaction with CI values of 0, 1 line showed R type reaction with a CI value of 4, 10 lines expressed MR reaction with CI values between 2 and 8, 69 lines showed M type reaction with CI values between 3 and 24, and 6 lines exhibited MS reaction with a CI value between 24 and 48, while Morocco showed S type reaction with a CI value of 100. Likewise, 11 lines showed 0 type reactions with CI values of 0, 11 lines exhibited R type reaction with CI values of 1, 11 lines expressed MR reaction with CI values between 4 and 16, 4 lines displayed MS reaction with CI values between 24 and 40, and 55 lines expressed M type reactions with CI values between 3 and 24, while Morocco showed S reaction with a CI value of 90 at Peshawar.

**Figure 2:**
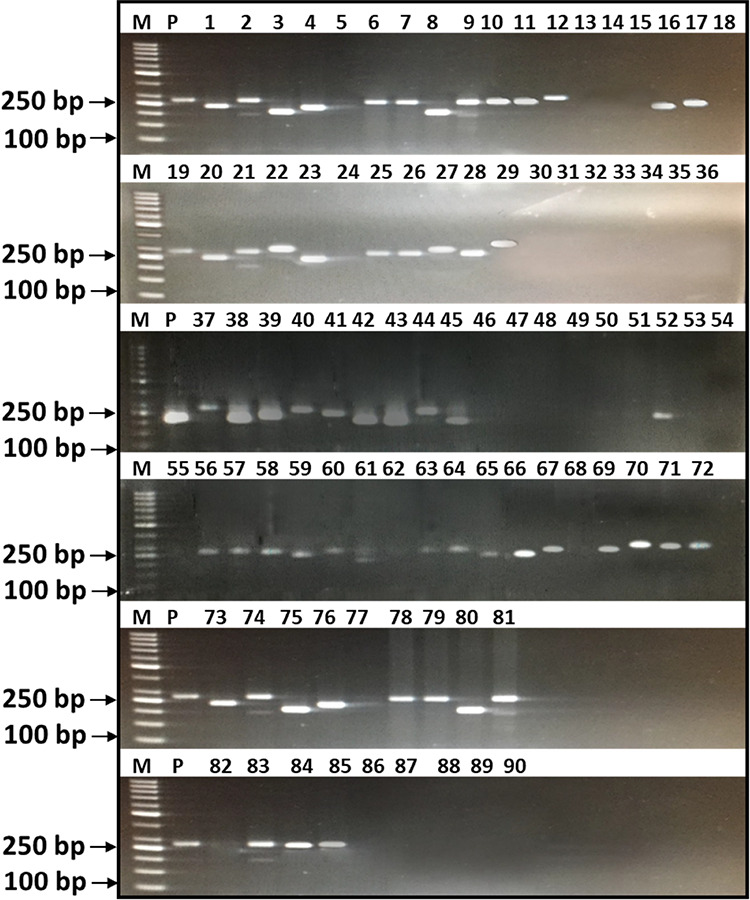
PCR amplification profile of *Yr10*, separated on 2% agarose gel. M: 269bp, DNA ladder: 50bp, P: positive, 1–90 show genotypes.

**Figure 3:**
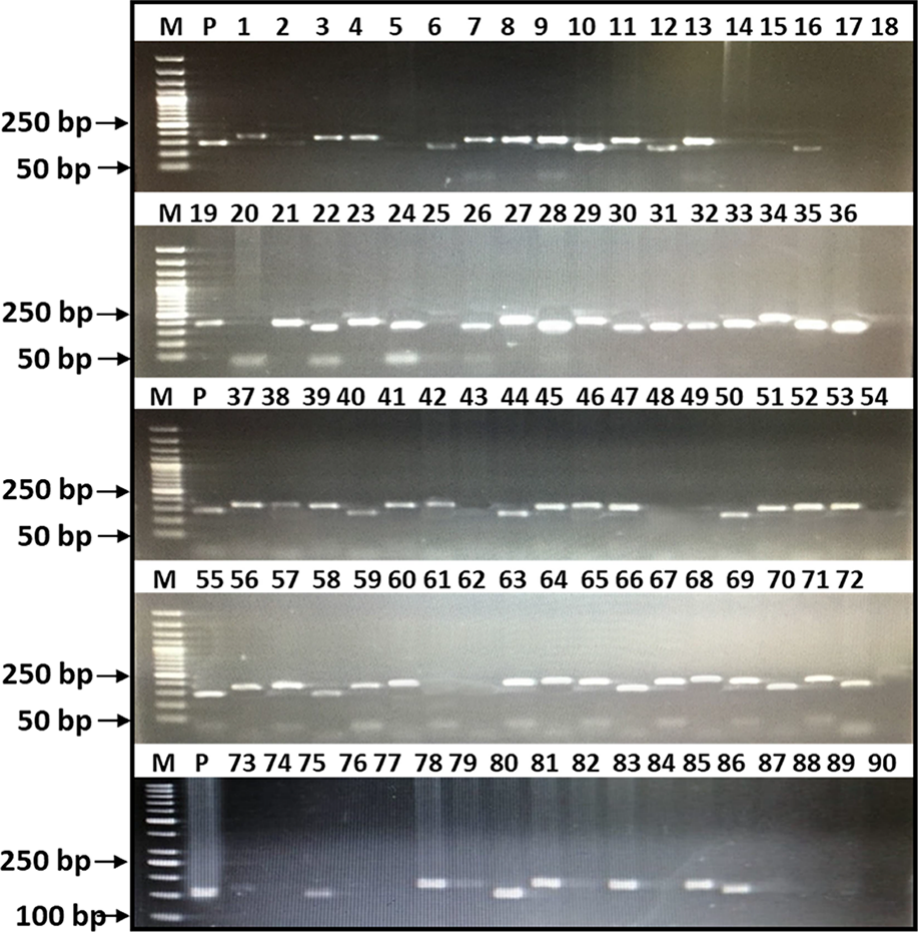
PCR amplification profile of the *Yr15* gene, separated on 2% agarose gel. M: 190bp, DNA ladder: 50 bp, P: positive, 1–90 show genotypes.

SSR marker analysis revealed that 16 lines including check cv. Morocco showed absence of both *Yr15* and *Yr10* genes, 17 lines including check cv. Kohat-2017 expressed the presence of the *Yr15* gene only, 9 lines including Pakistan-2013 carries the *Yr10* gene only, while 49 lines carry both *Yr15* and *Yr10* genes (Figures 2 and 3). Combining the molecular phenotypic data, among top 25 lines based on ACI values, nine lines carrying both *Yr15* and *Yr10* genes showed high resistance to stripe rust exhibiting lower ACI values, while nine lines carrying *Yr15* and three lines carrying only the *Yr10* gene only also showed desirable lower ACI values indicating effectiveness of these genes against stripe rust. Surprisingly, four lines showed desirable lower ACI values without carrying *Yr15* and *Yr10*, showing the presence of unique genes in their genetic makeup.

## 4 Discussion

Stripe rust, often known as stripe rust, is a damaging wheat disease brought on by *P. striiformisf. sp. tritici* (Pst), which, in extreme cases, can cause a full loss of yield. East Africa, Australia, and North America (especially the Pacific Northwest), East Asia, and South Asia have all recently been the subject of reports of local epidemics.^44^ Further study is needed to find and characterize new resistance genes that may be employed to slow down the degradation of the resistance that is already present in wheat germplasm due to the increased incidence of Pst and the dearth of disease resistance genes in wheat.^43−45^ In the 2018–2019 growing season, 93 spring wheat lines and the 3 check varieties Kohat-2017, Pakistan-2013, and Morocco were assessed besides agricultural characteristics and disease tolerance in the Barani Agricultural Research Station, Kohat, the IBGE, Peshawar, and the Cereal Crops Research Institute, Pirsabak, Nowshera. Grain yield in wheat is influenced by a number of agronomic and physiological variables that have been thoroughly investigated and analyzed in wheat development initiatives.^46,47^ Due to their high heritability and connection to grain yield, agronomic and morphological traits can be used as indirect selection criteria during breeding and cultivar development.^46−49^ Yet, it has been proposed that a single variety’s multiple characteristics that confer improved agronomic and physiological performance as well as biotic and abiotic stress tolerance can be simultaneously selected and introgressed. This would result in a genetic improvement in final yield.^50^ Our research showed that genotypes behaved differently in various contexts, demonstrating the importance of environmental influences on the expression of phenotypic traits. For practically all variables at Kohat, Nowshera, and Peshawar, significant variations were observed in the ANOVA (Analysis of Variance) across genotypes, lines, checks, and checks and lines. Throughout the course of a only one growth phase, variations in field parameters such as warmth, rainfall, moisture, and the timing of cultivation, nitrogen fertilizer, infectious frequencies, and soil composition of diseases at various sites have an impact on wheat output and quality.^51^ Although some genotypes function consistently, others show significant environmental heterogeneity.^52^ In comparison to controls, genetic advances estimated through yield trials vary among contemporary varieties released at various dates. Agronomic and physiological factors that are connected to yield have the biggest effects on genetic gain variation.^50,53−56^ The findings of the current investigation demonstrate that grain yield significantly adversely correlated with 1000-grain weight but positively with grain spike^–1^, grain weight spike^–1^, dates till maturity, time to bearing, and seed. In contrast to Nowshera, days to maturity had a substantial positive connection of seed spike^–1^, kernel mass spike^–1^, and tillers m^–2^ but a sizable detrimental association of grain yield. In Peshawar, grain output and thousand seed mass, plant length, spike mass, as well as tillers m^–2^ all showed significant positive correlations. It has been demonstrated that an increase in thousand-grain weight significantly correlates with an increase in grain production.^57,58^ The selection response for this feature in low-yielding situations was, however, constrained by a nonsignificant contribution of 1000-grain weight that was seen, particularly under heat stress circumstances.^50,53,59−61^ Despite the fact that the genotypes of wheat used in our study were the same in all three locations, G × E interaction may be responsible for some of the variation in line characteristics. Since 1948, Pakistan has had 13 epidemics of the bread wheat disease stripe rust.^62^ The worst epidemic during the 2003 and 2004 wheat seasons was that of Inqilab-91, a local bread wheat type, which badly damaged 80% of the nation’s wheat-growing territory.^63^ Infection rates were said to be higher in the northern regions and lower in the western and central highlands of the nation. Due to the dry and hot climate, stripe rust used to happen in the southern regions of the nation. Pst has recently had an impact on wheat in the southern regions of the provinces of Punjab and Sindh.^64^

Although stripe rust is a serious foliar disease of bread wheat in Pakistan and causes large losses every year, nothing is known about the infecting strain’s pathogenicity.^65−67^ To control the disease and avoid yield loss, it is crucial to assess the virulence of Pst populations for the introduction of potential resistance genes in the elite types. New races of the rust pathogen emerge in a few years even after the resistant gene is inserted into superior germplasm, destroying the resistance and resulting in severe stripe rust epidemics. For instance, *Yr27* had been a frequently employed resistance source in wheat cultivars from 2002 to 2004 before becoming susceptible as a result of the introduction of fresh pathogenic races in Pakistan and India.^31,67^ In China, Iran, Pakistan, and the USA, the *Yr10* gene has been reported to be racial specific and effective against all races.^68−70^ In our investigation, 16 lines, including the check cv, were found using SSR marker analysis. In 17 lines, including the check cv, the *Yr5* and *Yr10* genes were absent in Morocco. Only the *Yr15* gene was present in Kohat-2017 but the *Yr10* gene was present in 9 lines, including Pakistan-2013 and 49 genotypes. Using molecular phenotypic data, nine lines with both the *Yr15* and *Yr10* genes were found to be resistant to stripe rust with reduced ACI readings, while nine lines without both *Yr15* and *Yr10* genes did the same as lines with just the *Yr15* gene; three lines with just the *Yr10* gene demonstrated desirable lower ACI values, demonstrating the potency of these genes against stripe rust. Notably, *Yr15* and *Yr10* were excluded from the four lines with acceptable lower ACI values, demonstrating that these lines have distinct genetic makeup and discrete genes.

Based on the above discussion, it can be concluded that significant differences among genotypes were observed for almost all traits at all three sites with few exceptions exhibiting enough variability among existing wheat germplasms. Grain yield showed positive and significant correlation with grain spike^–1^, grain weight spike^–1^, tillers m^–2^, 1000-grain weight, spike weight, and tillers m^–2^. Genotype KT-72, KT-07, and KT-10 produced maximum grain yield with a desirable low ACI value carrying both *Yr*15 and *Yr*10 genes in their genetic makeup showing yield potential and stripe rust resistance for exploitation in the future wheat breeding program.

## Data Availability

All data generated or analyzed during this study are included in this published article.

## Supporting Information Available

Mean values for various traits of 93 wheat genotypes at Kohat (E-01), Nowshera (E-02), and Peshawar (E-03) during 2018–2019 (PDF)

## Notes

A set of 93 advance bread wheat lines including 3 checks varieties such as Morocco, Kohat-2017, and Pakistan-2013 were field tested at three locations of Khyber Pakhtunkhwa, that is, Barani Agricultural Research Station in Kohat (E-01), the Cereal Crops Research Institute in Pirsabak (E-02), and the Department of Biotechnology and Genetic Engineering at the University of Agriculture in Peshawar (E-03). All the lab activities of the project were conducted at the IBGE of University of Agriculture Peshawar. Every experiment complies with all applicable laws and rules.
