# Supplementary material for: Genetic Characterization of Advance Bread Wheat Lines for Yield and Stripe Rust Resistance
Source: ACS Omega. 2023 Jul 11;8(29):25988–98. doi: 10.1021/acsomega.3c01981 (PMC10372943; doi:10.1021/acsomega.3c01981)
Supplement: Supplementary file 1 — ao3c01981_si_001.pdf [file ao3c01981_si_001.pdf]

Genetic Characterization of Advance Bread Wheat Lines for Yield and Stripe Rust Resistance

Israr Ud Din [†1], Salman Khan [†2], Fahim Ullah Khan [†3*], Majid Khan [1], Muhammad Nauman Khan [4, 5], Aqsa Hafeez [6], Sana Wahab [6], Nazima Wahid [6], Baber Ali [6*], Umair Bin Qasim [7], Fazal Manan [8], Mona S. Alwahibi [9], Mohamed S. Elshikh [9], Sezai Ercisli [10, 11*], Ebaa Mohamed Ali Khalifa [12]

1. The University of Agriculture Peshawar, Institute of Biotechnology and Genetic Engineering; Peshawar, PK 25130; israr.uddin@aup.edu.pk; majid_ibge@yahoo.com
2. Abdul Wali Khan University Mardan, Department of Biotechnology; Mardan, PK 23200; salmankhan32150@yahoo.com
3. Hazara University, Department of Agriculture; Mansehra, PK 21120; fahimbiotech@hu.edu.pk
4. Islamia College Peshawar, Department of Botany; Peshawar, PK 25120; nomiflora@uop.edu.pk
5. University Public School, University of Peshawar, 25120 Peshawar, Pakistan
6. Quaid-i-Azam University, Department of Plant Sciences, Islamabad, 45320, PK; aqsahafeez@bs.qau.edu.pk; sanawahab1995@gmail.com (S.W); nazimasoomro1@gmail.com: baberali@bs.qau.edu.pk (B.A)
7. The University of Agriculture Peshawar, Department of Plant Breeding & Genetics; Peshawar, PK 25130; umairkhan9075726@gmail.com (U.B.Q)
8. North Dakota State University, Department of Plant Pathology, Fargo, ND, USA 58108-6050; fazal.manan@ndsu.edu
9. King Saud University, Department of Botany and Microbiology, College of Science, Riyadh, 11451, SA; malwhibi@ksu.edu.sa; melshikh@ksu.edu.sa
10. Ataturk University, Department of Horticulture Faculty of Agriculture, Erzurum, 25240, TR; sercisli@atauni.edu.tr
11. HGF Agro, Ata Teknokent, TR-25240 Erzurum, Turkey
12. Agriculture Research Center, Wheat Research Department, Field Crop Research Institute, Giza, EG 3725005; Eba_khalifa@yahoo.com

[†]These authors contributed equally to this work

[*]Corresponding author: fahimbiotech@hu.edu.pk, baberali@bs.qau.edu.pk; sercisli@atauni.edu.tr

Supporting Information

The data represents the Mean values for various traits of 93 wheat genotypes at Kohat (E-01), Nowshera (E-02) and Peshawar (E-03) during 2018-19.

Table S1. Mean values for tillers m^{-2} of 93 wheat genotypes at Kohat (E-01), Nowshera (E-02) and Peshawar (E-03) during 2018-19.

Genotype	E-01	E-02	E-03	Mean	Genotype	E-01	E-02	E-03	Mean
KT-01	167	205	182	185	KT-48	233	196	135	188
KT-02	129	222	150	167	KT-49	222	172	129	174
KT-03	108	219	169	166	KT-50	157	170	177	168
KT-04	199	226	150	192	KT-51	233	160	176	189
KT-05	144	397	403	315	KT-52	219	202	155	192
KT-06	108	309	213	210	KT-53	253	184	262	233
KT-07	230	240	184	218	KT-54	155	241	147	181
KT-08	173	197	115	162	KT-55	135	218	149	167
KT-09	252	387	75	238	KT-56	179	215	210	201
KT-10	191	293	304	262	KT-57	178	429	233	280
KT-11	136	335	159	210	KT-58	253	206	197	219

KT-12	177	250	203	210	KT-59	261	294	226	261
KT-13	147	204	135	162	KT-60	167	261	104	178
KT-14	125	322	249	232	KT-61	151	326	192	223
KT-15	144	201	179	175	KT-62	238	334	193	255
KT-16	168	305	346	273	KT-63	181	325	175	227
KT-17	200	225	140	188	KT-64	133	171	240	181
KT-18	79	324	181	195	KT-65	159	296	173	210
KT-19	134	392	188	238	KT-66	168	186	88	147
KT-20	117	345	149	204	KT-67	143	328	180	217
KT-21	175	379	144	232	KT-68	137	310	154	200
KT-22	100	275	229	201	KT-69	164	217	254	212
KT-23	205	185	118	169	KT-70	197	166	164	176

KT-24	124	238	149	171	KT-71	259	195	121	192
KT-25	224	282	168	225	KT-72	170	280	351	267
KT-26	78	95	124	99	KT-73	188	572	202	321
KT-27	145	215	153	171	KT-74	197	327	238	254
KT-28	141	313	134	196	KT-75	202	254	223	227
KT-29	137	277	149	188	KT-76	201	324	153	226
KT-30	134	195	133	154	KT-77	140	259	255	218
KT-31	154	230	153	179	KT-78	261	205	138	202
KT-32	300	134	244	226	KT-79	196	181	251	209
KT-33	253	344	157	251	KT-80	107	428	236	257
KT-34	219	263	170	217	KT-81	176	538	203	305
KT-35	178	187	194	186	KT-82	137	196	283	205

KT-36	261	216	130	202	KT-83	139	165	167	157
KT-37	307	256	208	257	KT-84	142	196	184	174
KT-38	222	172	213	203	KT-85	112	298	288	233
KT-39	194	183	255	211	KT-86	197	626	152	325
KT-40	172	148	222	181	KT-87	172	226	204	201
KT-41	216	336	151	234	KT-88	201	372	232	268
KT-42	190	241	207	213	KT-89	121	309	302	244
KT-43	193	247	198	213	KT-90	124	292	182	199
KT-44	273	253	231	252	Morroco	110	221	142	157
KT-45	146	148	232	175	Kohat-17	140	172	178	164
KT-46	170	121	144	145	Pakistan-13	158	215	187	187
KT-47	271	243	207	240	--	--	--	--	--

Mean					CD(0.05)				
Genotypes	177	261	190			--	--	--	--
Checks(C)	136	203	169			15.59	59.41	56.07	
Lines	179	263	191			27.00	102.90	97.12	
C vs. L	--					22.05	84.02	79.30	

Table S2. **Mean values for spike weight of 93 wheat genotypes at Kohat (E-01), Nowshera (E-02) and Peshawar (E-03) during 2018-19.**

Genotype	E-01	E-02	E-03	Mean	Genotype	E-01	E-02	E-03	Mean
KT-01	3.5	2.5	5.1	3.71	KT-48	6.33	3.3	4.4	4.7
KT-02	3.1	3.5	4.5	3.71	KT-49	3.33	3.7	4.0	3.7
KT-03	3.9	3.1	3.5	3.51	KT-50	5.73	3.9	4.0	4.6
KT-04	3.5	3.3	3.9	3.57	KT-51	3.93	3.3	5.0	4.1
KT-05	3.1	3.1	3.5	3.24	KT-52	3.33	2.7	4.2	3.4
KT-06	3.1	2.3	3.3	2.91	KT-53	3.33	3.1	3.8	3.4
KT-07	3.5	3.5	4.7	3.91	KT-54	4.33	2.9	3.8	3.7
KT-08	3.9	3.1	4.7	3.91	KT-55	6.93	2.7	4.6	4.8
KT-09	3.1	1.9	3.9	2.97	KT-56	3.93	2.1	3.8	3.3
KT-10	3.9	3.3	3.5	3.57	KT-57	3.73	1.7	3.6	3.0
KT-11	3.7	2.3	3.1	3.04	KT-58	4.93	2.3	3.2	3.5

KT-12	3.5	3.3	3.9	3.57	KT-59	4.53	2.5	3.4	3.5
KT-13	3.1	3.5	4.3	3.64	KT-60	4.13	3.3	4.0	3.8
KT-14	4.3	2.1	2.7	3.04	KT-61	5.14	2.8	3.3	3.7
KT-15	3.5	3.7	3.7	3.64	KT-62	5.14	3.0	3.5	3.9
KT-16	3.9	2.5	3.1	3.17	KT-63	3.14	3.4	3.7	3.4
KT-17	2.7	2.9	4.7	3.44	KT-64	4.34	2.8	2.9	3.3
KT-18	3.5	2.7	3.9	3.37	KT-65	3.14	2.8	3.7	3.2
KT-19	3.1	2.3	2.7	2.71	KT-66	2.74	2.4	4.3	3.1
KT-20	3.7	2.5	3.3	3.17	KT-67	3.14	2.6	3.7	3.1
KT-21	3.9	2.1	4.3	3.44	KT-68	3.54	2.4	3.7	3.2
KT-22	3.3	3.1	3.9	3.44	KT-69	2.94	3.0	3.7	3.2
KT-23	3.5	3.3	4.3	3.71	KT-70	4.14	3.2	4.1	3.8

KT-24	2.7	3.9	3.5	3.37	KT-71	4.54	3.0	3.3	3.6
KT-25	3.3	3.1	4.3	3.57	KT-72	3.94	3.0	3.3	3.4
KT-26	4.7	3.9	2.3	3.64	KT-73	3.74	2.2	3.5	3.1
KT-27	3.9	3.7	3.9	3.84	KT-74	3.94	2.8	2.9	3.2
KT-28	2.7	2.9	3.7	3.11	KT-75	6.74	3.0	3.1	4.3
KT-29	4.7	3.1	3.5	3.77	KT-76	5.74	2.6	2.7	3.7
KT-30	3.3	3.7	3.7	3.57	KT-77	6.14	2.4	2.9	3.8
KT-31	4.1	3.3	3.8	3.76	KT-78	3.34	3.4	3.7	3.5
KT-32	5.5	3.7	3.8	4.36	KT-79	5.54	3.0	3.1	3.9
KT-33	5.3	2.3	4.2	3.96	KT-80	3.54	1.8	3.1	2.8
KT-34	3.5	3.9	4.4	3.96	KT-81	3.34	1.6	2.5	2.5
KT-35	3.7	3.5	3.2	3.50	KT-82	3.14	2.4	2.7	2.7

KT-36	3.1	2.7	4.2	3.36	KT-83	3.74	2.2	2.9	2.9
KT-37	2.9	2.7	2.8	2.83	KT-84	6.54	2.8	3.3	4.2
KT-38	3.5	3.1	3.4	3.36	KT-85	3.94	3.0	3.5	3.5
KT-39	3.7	3.1	2.8	3.23	KT-86	3.34	2.6	4.1	3.3
KT-40	3.1	4.1	3.4	3.56	KT-87	6.74	3.4	3.5	4.5
KT-41	4.7	2.1	3.8	3.56	KT-88	3.34	2.0	2.9	2.7
KT-42	4.7	3.1	3.4	3.76	KT-89	3.74	3.0	4.3	3.7
KT-43	4.1	2.9	3.4	3.50	KT-90	4.34	2.9	3.5	3.6
KT-44	3.9	3.3	3.6	3.63	Morroco	2.79	2.5	2.9	2.7
KT-45	4.3	3.1	3.2	3.56	Kohat-17	3.66	4.3	3.7	3.9
KT-46	6.9	2.9	4.0	4.63	Pakistan-13	2.89	3.4	3.7	3.3
KT-47	6.1	2.7	3.0	3.96	--	--	--	--	--

Mean					CD(0.05)				
Genotypes	4.0	2.9	3.6			--	--	--	--
Checks(C)	3.1	3.4	3.4			0.42	0.59	0.52	
Lines	4.1	2.9	3.6			0.72	1.02	0.90	
C vs. L	--					0.59	0.83	0.74	

Table S3. Mean values for grains weight spike^{-1} of 93 wheat genotypes at Kohat (E-01), Nowshera (E-02) and Peshawar (E-03) during 2018-19.

Genotype	E-01	E-02	E-03	Mean	Genotype	E-01	E-02	E-03	Mean
KT-01	3.05	1.64	2.87	2.52	KT-48	2.04	2.26	2.67	2.33
KT-02	2.16	2.00	3.07	2.41	KT-49	1.84	2.55	2.07	2.15
KT-03	2.16	1.56	1.87	1.86	KT-50	3.69	2.82	2.07	2.86
KT-04	1.55	2.00	2.07	1.88	KT-51	1.90	2.95	2.67	2.51
KT-05	1.75	1.34	1.67	1.59	KT-52	2.06	2.15	2.27	2.16
KT-06	1.92	1.16	1.87	1.65	KT-53	1.79	2.34	2.07	2.07
KT-07	1.99	2.00	2.47	2.15	KT-54	2.32	1.96	2.27	2.18
KT-08	1.87	1.99	2.47	2.11	KT-55	2.42	1.81	2.67	2.30
KT-09	1.21	1.24	2.07	1.51	KT-56	2.14	1.45	1.67	1.75
KT-10	1.65	1.82	1.87	1.78	KT-57	1.97	1.27	1.67	1.64
KT-11	1.77	1.43	1.87	1.69	KT-58	2.29	1.55	1.67	1.84

KT-12	1.80	2.17	2.27	2.08	KT-59	1.53	1.63	1.27	1.47
KT-13	2.66	2.38	2.67	2.57	KT-60	2.59	2.13	3.07	2.60
KT-14	2.57	1.50	1.67	1.91	KT-61	2.72	1.67	1.86	2.08
KT-15	2.24	2.66	2.27	2.39	KT-62	2.16	1.36	1.86	1.79
KT-16	2.03	1.51	1.27	1.60	KT-63	2.21	1.59	1.86	1.88
KT-17	2.20	2.36	2.67	2.41	KT-64	2.24	1.46	1.26	1.65
KT-18	2.70	1.37	2.27	2.11	KT-65	2.10	1.39	2.06	1.85
KT-19	2.04	1.19	1.67	1.63	KT-66	2.43	1.58	2.86	2.29
KT-20	2.00	1.53	2.07	1.87	KT-67	1.66	1.21	1.46	1.44
KT-21	1.80	1.34	2.47	1.87	KT-68	1.31	0.97	2.66	1.65
KT-22	3.65	1.85	2.27	2.59	KT-69	1.72	1.65	2.06	1.81
KT-23	1.86	2.17	2.27	2.10	KT-70	2.08	1.70	2.06	1.94

KT-24	2.02	2.20	2.07	2.10	KT-71	1.25	1.32	2.26	1.61
KT-25	1.75	1.78	1.87	1.80	KT-72	2.80	1.69	1.46	1.98
KT-26	2.28	2.02	1.87	2.06	KT-73	1.97	0.60	2.06	1.54
KT-27	2.38	2.22	2.47	2.36	KT-74	1.71	1.45	1.86	1.67
KT-28	2.10	1.51	2.47	2.03	KT-75	2.22	1.47	1.66	1.78
KT-29	3.05	1.69	2.07	2.27	KT-76	1.69	1.06	2.06	1.60
KT-30	2.16	2.54	1.87	2.19	KT-77	1.53	1.32	1.66	1.50
KT-31	2.20	2.22	1.67	2.03	KT-78	1.61	1.80	2.06	1.82
KT-32	1.59	2.87	1.67	2.04	KT-79	2.04	1.40	2.06	1.83
KT-33	1.79	1.57	2.47	1.94	KT-80	2.61	0.25	1.66	1.51
KT-34	1.96	1.34	2.47	1.92	KT-81	1.79	0.52	1.46	1.26
KT-35	2.03	2.34	1.27	1.88	KT-82	1.83	1.42	1.46	1.57

KT-36	1.91	1.92	2.27	2.04	KT-83	1.88	1.18	1.66	1.57
KT-37	1.25	2.02	1.27	1.52	KT-84	1.43	1.76	1.86	1.68
KT-38	1.69	1.67	1.47	1.61	KT-85	1.63	1.64	2.06	1.78
KT-39	1.81	2.16	1.07	1.68	KT-86	1.79	0.71	2.66	1.72
KT-40	1.84	2.77	1.87	2.16	KT-87	1.90	1.72	1.86	1.82
KT-41	2.70	1.31	2.07	2.03	KT-88	1.70	1.13	1.26	1.36
KT-42	2.61	2.27	1.87	2.25	KT-89	1.69	1.54	1.66	1.63
KT-43	2.28	1.84	1.87	2.00	KT-90	1.98	1.55	2.06	1.86
KT-44	2.07	1.67	1.87	1.87	Morroco	1.67	1.50	1.93	1.70
KT-45	2.24	2.33	1.47	2.02	Kohat-17	2.44	2.51	2.33	2.43
KT-46	2.77	2.71	2.07	2.52	Pakistan-13	1.97	1.93	2.26	2.06
KT-47	2.08	2.02	1.67	1.93	--	--	--	--	--

Mean					CD(0.05)				
Genotypes	2.06	1.74	2.01			--	--	--	--
Checks(C)	2.03	1.98	2.17			0.11	0.50	0.53	
Lines	2.06	1.73	2.00			0.20	0.86	0.92	
C vs. L	--					0.16	0.70	0.75	

Table S4. Mean values for grains spike^{-1} of 93 wheat genotypes at Kohat (E-01), Nowshera (E-02) and Peshawar (E-03) during 2018-19.

Genotype	E-01	E-02	E-03	Mean	Genotype	E-01	E-02	E-03	Mean
KT-01	70	47	76	64	KT-48	44	69	75	63
KT-02	53	61	86	67	KT-49	38	72	50	54
KT-03	47	43	55	48	KT-50	89	81	51	74
KT-04	40	63	58	54	KT-51	44	87	64	65
KT-05	38	39	44	40	KT-52	49	59	57	55
KT-06	45	35	45	42	KT-53	39	66	53	53
KT-07	49	57	64	56	KT-54	55	59	64	59
KT-08	49	55	77	60	KT-55	54	51	77	61
KT-09	30	32	57	39	KT-56	49	43	50	47
KT-10	37	50	48	45	KT-57	48	36	44	43
KT-11	45	38	46	43	KT-58	56	42	47	49

KT-12	42	64	57	54	KT-59	40	45	40	41
KT-13	56	67	69	64	KT-60	62	63	78	67
KT-14	68	46	47	54	KT-61	66	52	60	59
KT-15	53	72	54	60	KT-62	55	43	57	52
KT-16	47	40	37	41	KT-63	55	45	54	52
KT-17	57	63	75	65	KT-64	54	45	40	46
KT-18	58	35	58	51	KT-65	49	42	59	50
KT-19	47	35	51	45	KT-66	64	50	74	62
KT-20	47	47	53	49	KT-67	40	39	46	42
KT-21	48	39	62	49	KT-68	33	30	65	43
KT-22	75	54	58	63	KT-69	40	51	67	53
KT-23	45	66	56	56	KT-70	50	51	59	53

KT-24	47	66	58	57	KT-71	31	41	68	47
KT-25	46	52	52	50	KT-72	67	54	44	55
KT-26	53	57	58	56	KT-73	45	21	57	41
KT-27	60	60	65	62	KT-74	41	45	54	47
KT-28	51	41	67	53	KT-75	55	45	53	51
KT-29	65	49	52	55	KT-76	43	34	55	44
KT-30	45	70	48	54	KT-77	33	42	48	41
KT-31	49	67	44	53	KT-78	40	55	56	50
KT-32	34	79	47	53	KT-79	51	45	59	51
KT-33	42	45	61	49	KT-80	63	10	54	42
KT-34	44	38	59	47	KT-81	45	17	46	36
KT-35	43	60	38	47	KT-82	49	42	46	45

KT-36	42	50	57	50	KT-83	47	38	50	45
KT-37	26	52	41	40	KT-84	34	54	55	48
KT-38	40	49	46	45	KT-85	42	50	56	50
KT-39	41	59	34	45	KT-86	45	21	69	45
KT-40	43	73	48	55	KT-87	46	55	48	50
KT-41	64	36	54	51	KT-88	42	36	41	39
KT-42	56	69	51	59	KT-89	43	46	48	46
KT-43	47	55	50	51	KT-90	47	47	59	51
KT-44	45	48	47	47	Morroco	51	49	61	54
KT-45	51	70	42	54	Kohat-17	61	41	51	51
KT-46	68	72	60	67	Pakistan-13	51	39	56	49
KT-47	47	58	44	50	--	--	--	--	--

Mean					CD(0.05)				
Genotypes	49	50	55			--	--	--	--
Checks(C)	54	43	56			4.25	9.81	12.89	
Lines	49	50	55			7.36	16.99	22.33	
C vs. L	--					6.01	13.87	18.23	

Table S5. Mean values for 1000 grain weight of 93 wheat genotypes at Kohat, Nowshera and Peshawar during 2018-19.

Genotype	E-01	E-02	E-03	Mean	Genotype	E-01	E-02	E-03	Mean
KT-01	43.3	34.8	37.9	38.6	KT-48	46.4	31.9	36.4	38.2
KT-02	40.9	32.9	35.7	36.5	KT-49	48.4	34.9	41.5	41.6
KT-03	46.2	36.1	33.7	38.6	KT-50	41.5	34.3	40.9	38.9
KT-04	39.1	31.9	35.4	35.4	KT-51	42.9	33.2	42.2	39.4
KT-05	46.6	33.9	37.7	39.4	KT-52	42.1	35.9	40.1	39.4
KT-06	42.3	33.0	40.9	38.7	KT-53	46.1	35.1	39.4	40.2
KT-07	40.8	35.1	38.9	38.2	KT-54	42.4	32.2	36.4	37.0
KT-08	38.5	36.4	32.1	35.6	KT-55	45.1	35.1	35.5	38.6
KT-09	40.9	37.9	36.5	38.4	KT-56	43.8	33.2	34.1	37.0
KT-10	45.2	36.1	38.5	39.9	KT-57	41.1	34.2	38.9	38.1
KT-11	39.1	36.8	40.5	38.8	KT-58	41.0	35.8	36.3	37.7

KT-12	42.9	34.0	39.9	38.9	KT-59	38.3	35.6	33.2	35.7
KT-13	47.5	35.5	38.7	40.5	KT-60	41.7	33.4	40.1	38.4
KT-14	37.5	32.7	35.5	35.2	KT-61	41.3	33.0	29.8	34.7
KT-15	42.3	37.2	41.7	40.4	KT-62	39.0	32.8	32.0	34.6
KT-16	42.8	37.8	33.9	38.1	KT-63	39.7	35.9	33.9	36.5
KT-17	38.8	37.2	35.9	37.3	KT-64	41.1	33.8	30.4	35.1
KT-18	46.2	38.8	38.9	41.3	KT-65	42.6	33.8	34.3	36.9
KT-19	43.2	33.1	32.5	36.2	KT-66	38.2	32.8	37.8	36.3
KT-20	42.2	32.0	38.7	37.6	KT-67	40.9	32.5	30.8	34.8
KT-21	37.4	34.4	40.1	37.3	KT-68	39.1	33.8	40.1	37.7
KT-22	48.5	33.9	38.9	40.4	KT-69	42.5	33.3	29.9	35.3
KT-23	41.2	32.8	40.5	38.1	KT-70	41.6	34.4	34.3	36.8

KT-24	43.1	33.4	35.5	37.3	KT-71	40.1	33.3	32.3	35.3
KT-25	38.3	34.4	35.5	36.0	KT-72	41.6	32.3	32.7	35.6
KT-26	42.8	35.4	31.9	36.7	KT-73	43.1	31.8	35.3	36.8
KT-27	39.7	37.3	37.9	38.3	KT-74	41.4	32.9	34.0	36.1
KT-28	41.3	36.8	37.1	38.4	KT-75	40.1	33.3	30.7	34.7
KT-29	46.6	34.6	39.9	40.3	KT-76	39.0	32.7	36.8	36.2
KT-30	48.6	36.4	39.1	41.3	KT-77	45.4	32.5	34.1	37.4
KT-31	44.9	32.5	38.5	38.6	KT-78	40.2	33.7	35.8	36.6
KT-32	47.4	35.8	36.5	39.9	KT-79	40.1	32.3	34.4	35.6
KT-33	43.1	34.4	40.8	39.4	KT-80	41.1	32.5	29.8	34.5
KT-34	45.3	34.0	42.1	40.5	KT-81	39.9	33.7	30.7	34.8
KT-35	48.1	38.8	34.1	40.3	KT-82	37.6	35.0	31.3	34.7

KT-36	46.4	37.9	40.5	41.6	KT-83	39.7	32.3	32.1	34.7
KT-37	49.5	38.8	31.9	40.1	KT-84	42.0	33.4	33.0	36.2
KT-38	42.2	33.3	33.2	36.2	KT-85	38.2	33.5	35.8	35.9
KT-39	44.4	36.2	32.5	37.7	KT-86	39.2	36.1	37.8	37.7
KT-40	43.1	37.3	39.9	40.1	KT-87	41.0	31.9	38.0	37.0
KT-41	42.1	35.8	39.0	39.0	KT-88	40.6	33.3	29.8	34.6
KT-42	46.9	31.9	37.5	38.8	KT-89	38.8	34.7	33.8	35.8
KT-43	48.6	32.6	37.9	39.7	KT-90	42.1	34.0	34.1	36.7
KT-44	45.9	34.4	40.0	40.1	Morroco	32.7	30.8	31.5	31.6
KT-45	44.0	32.6	36.1	37.6	Kohat-17	40.0	61.2	46.3	49.1
KT-46	41.0	37.0	35.5	37.8	Pakistan-13	38.6	49.3	40.6	42.9
KT-47	44.4	34.4	38.9	39.2	--	--	--	--	--

Mean					CD(0.05)				
Genotypes	42.3	34.8	36.3			--	--	--	--
Checks(C)	37.1	47.1	39.5			1.27	2.06	3.59	
Lines	42.4	34.4	36.2			2.19	3.57	6.22	
C vs. L	--					1.79	2.92	5.08	

Table S6. Mean values for grain yield kg ha^{-1} of 93 wheat genotypes at Kohat (E-01), Nowshera (E-02) and Peshawar (E-03) during 2018-19.

Genotype	E-01	E-02	E-03	Mean	Genotype	E-01	E-02	E-03	Mean
KT-01	5140	3369	4462	4323	KT-48	2369	4460	2870	3233
KT-02	2806	4469	3995	3757	KT-49	2036	4482	2187	2902
KT-03	2362	3402	2579	2781	KT-50	2925	4949	3070	3648
KT-04	3084	4546	2579	3403	KT-51	2202	4916	3820	3646
KT-05	2528	5213	5379	4373	KT-52	2258	4327	2887	3157
KT-06	2084	3502	3229	2938	KT-53	2258	4371	4637	3755
KT-07	4584	4835	3829	4416	KT-54	1814	4616	2737	3055
KT-08	3251	3946	2445	3214	KT-55	1647	3860	3204	2904
KT-09	3028	4680	1329	3012	KT-56	1925	2949	3054	2642
KT-10	3140	5302	4595	4346	KT-57	1758	4927	3404	3363
KT-11	2417	4713	2429	3186	KT-58	5758	3049	2854	3887

KT-12	3195	5457	3829	4160	KT-59	3925	4538	2620	3694
KT-13	3973	4913	3095	3994	KT-60	4314	5471	2420	4068
KT-14	3251	4769	3329	3783	KT-61	4047	5671	2934	4217
KT-15	3251	5457	3395	4034	KT-62	5158	4905	2951	4338
KT-16	3417	4569	3395	3794	KT-63	3991	5427	2684	4034
KT-17	4417	5369	3212	4333	KT-64	2936	2505	2451	2630
KT-18	2195	4380	3429	3334	KT-65	3325	4382	2951	3553
KT-19	2751	4546	2512	3270	KT-66	4047	2938	2134	3040
KT-20	2362	5213	2562	3379	KT-67	2380	4349	2151	2960
KT-21	3140	5002	3012	3718	KT-68	1825	3427	3401	2884
KT-22	3751	5069	4312	4377	KT-69	2825	3627	4301	3584
KT-23	3806	4080	2279	3388	KT-70	4102	2760	2801	3221

KT-24	2528	5291	2562	3460	KT-71	3325	2671	2284	2760
KT-25	3917	5002	2562	3827	KT-72	4714	4894	4151	4586
KT-26	1806	2002	1895	1901	KT-73	3714	4427	3434	3858
KT-27	3473	4835	3195	3834	KT-74	3380	5038	3634	4017
KT-28	2973	4680	2812	3488	KT-75	4491	3894	3034	3806
KT-29	2084	4657	2562	3101	KT-76	3436	3849	2618	3301
KT-30	1417	5046	2029	2831	KT-77	2158	3649	3451	3086
KT-31	1702	5071	2204	2992	KT-78	4269	3682	2368	3440
KT-32	2369	4060	3570	3333	KT-79	3991	2582	4251	3608
KT-33	2258	5060	3154	3491	KT-80	2714	1927	3201	2614
KT-34	2147	3260	3437	2948	KT-81	3158	3760	2418	3112
KT-35	1814	4427	2237	2826	KT-82	2491	2849	3351	2897

KT-36	2480	4082	2404	2989	KT-83	2602	2038	2284	2308
KT-37	1897	5060	2404	3120	KT-84	2047	3427	2818	2764
KT-38	1869	2816	2787	2491	KT-85	1825	5082	4868	3925
KT-39	1758	3971	2570	2767	KT-86	3547	5505	3368	4140
KT-40	1591	4282	3570	3148	KT-87	3269	3927	3118	3438
KT-41	2925	4038	2595	3186	KT-88	3436	4705	2368	3503
KT-42	2480	5427	3320	3742	KT-89	2047	5005	4084	3712
KT-43	2202	4394	3170	3255	KT-90	2436	4443	3092	3324
KT-44	2814	4049	3720	3528	Morroco	1826	2739	2275	2280
KT-45	1647	3571	3037	2752	Kohat-17	3426	3542	3453	3473
KT-46	2369	3494	2470	2778	Pakistan-13	3111	3425	3433	3323
KT-47	2814	4816	3004	3544	--	--	--	--	--

Mean					CD(0.05)					
Genotypes	2909	4238	3058			--	--	--	--
Checks(C)	2788	3235	3054			225.54	404.10	557.13	
Lines	2913	4272	3058			390.64	699.92	964.98	
C vs. L						3186.9	571.49	787.90	
